# Neuronal cilia in energy homeostasis

**DOI:** 10.3389/fcell.2022.1082141

**Published:** 2022-12-08

**Authors:** Kathryn M. Brewer, Katlyn K. Brewer, Nicholas C. Richardson, Nicolas F. Berbari

**Affiliations:** ^1^ Department of Biology, Indiana University-Purdue University Indianapolis, Indianapolis, IN, United States; ^2^ Stark Neurosciences Research Institute, Indiana University, Indianapolis, IN, United States; ^3^ Center for Diabetes and Metabolic Diseases, Indiana University School of Medicine, Indianapolis, IN, United States

**Keywords:** Neuronal cilia, obesity, Leptin-melanocortin signaling, GPCR signaling, energy homeostasis

## Abstract

A subset of genetic disorders termed ciliopathies are associated with obesity. The mechanisms behind cilia dysfunction and altered energy homeostasis in these syndromes are complex and likely involve deficits in both development and adult homeostasis. Interestingly, several cilia-associated gene mutations also lead to morbid obesity. While cilia have critical and diverse functions in energy homeostasis, including their roles in centrally mediated food intake and peripheral tissues, many questions remain. Here, we briefly discuss syndromic ciliopathies and monogenic cilia signaling mutations associated with obesity. We then focus on potential ways neuronal cilia regulate energy homeostasis. We discuss the literature around cilia and leptin-melanocortin signaling and changes in ciliary G protein-coupled receptor (GPCR) signaling. We also discuss the different brain regions where cilia are implicated in energy homeostasis and the potential for cilia dysfunction in neural development to contribute to obesity. We close with a short discussion on the challenges and opportunities associated with studies looking at neuronal cilia and energy homeostasis. This review highlights how neuronal cilia-mediated signaling is critical for proper energy homeostasis.

## Introduction

**FIGURE 1:**
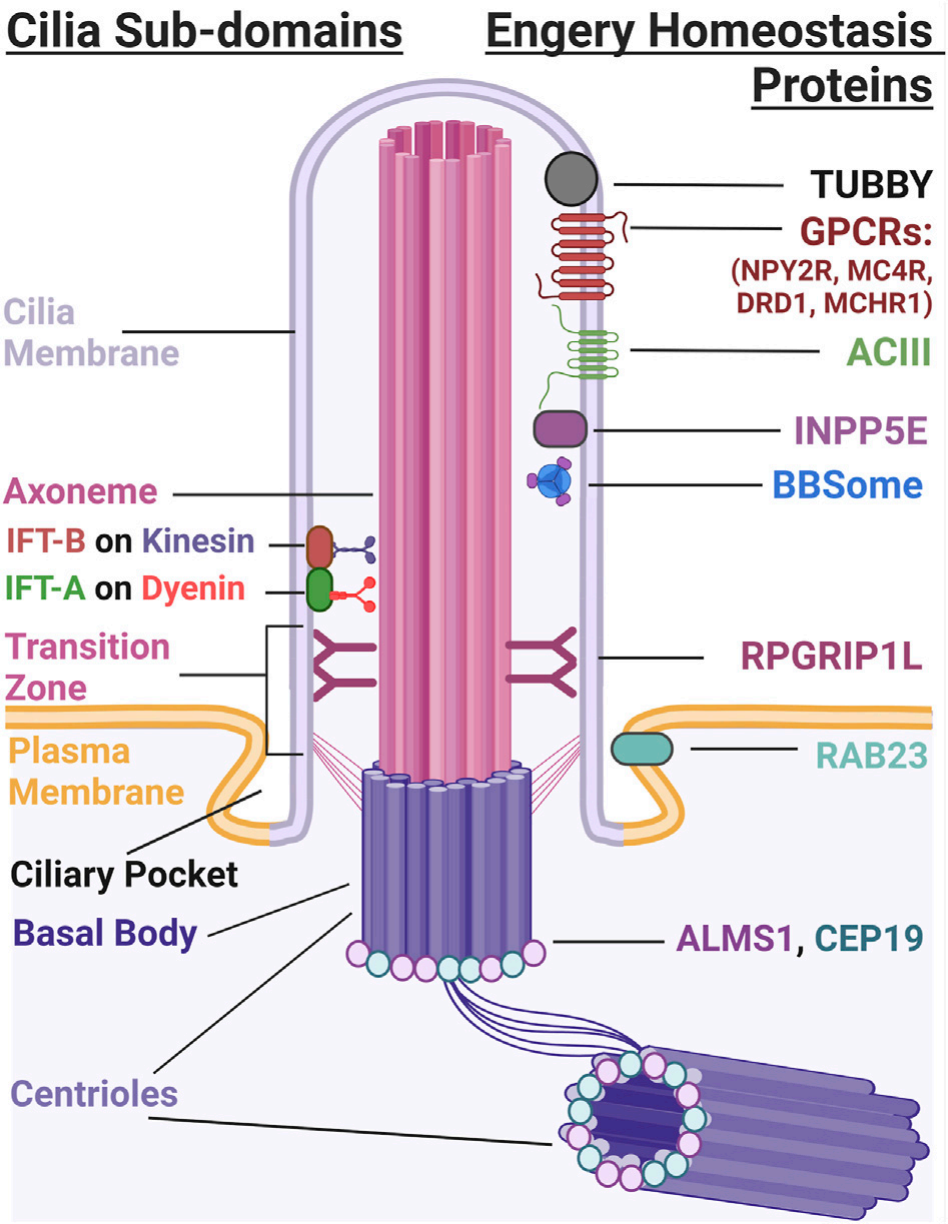
The Primary Cilium. The primary cilium has several subdomains that include the cilia membrane, axoneme, transition zone, ciliary pocket, and the basal body. IFT-A and IFT-B use dynein and kinesin motors to build and maintain cilia structure. Several different proteins localize to the cilium that are important for energy homeostasis. These include TUBBY (Mukhopadhyay and Jackson, 2013), GPCRs such as NPY2R, MCHR1, DRD1, and MC4R, ADCY3, and INPP5E which localize to the ciliary membrane. BBSome proteins traffic proteins to and from the ciliary membrane and RGRIP1L, RAB23, ALMS1 and CEP19 localize to the transition zone and base of the cilium, respectively.

Primary cilia are sensory, cellular appendages that regulate many signaling pathways (Wheway et al., 2018). Dysfunction of cilia leads to many pleiotropic syndromes collectively known as ciliopathies (Reiter and Leroux, 2017). Several ciliopathies such as Bardet-Biedl syndrome (BBS) and Alström Syndrome (ALMS) share a defining clinical feature of pediatric obesity. Additionally, there are many cilia-associated proteins, including G-protein coupled receptors (GPCRs) and signaling molecules [e.g., adenylyl cyclase 3 (ADCY3)], whose functions within the cilium are crucial for regulating energy homeostasis (Figure 1) (Vaisse et al., 2017; Engle et al., 2021). As obesity is a growing health concern, understanding how cilia regulate energy homeostasis and how their dysfunction contributes to this disease proves to be an important endeavor.

This review will briefly describe human ciliopathies associated with obesity and currently available data on clinical trials. We also describe genetic mouse models of ciliopathies and altered cilia-associated proteins that have led to new insights on mechanisms cilia use to regulate body weight. We will focus on the importance of neuronal cilia in regulating energy homeostasis by looking at conditional genetic mouse models which have implicated the hypothalamus and other brain regions. We also address potential neurodevelopmental roles for cilia in obesity, followed by a discussion suggesting that neuronal cilia within the hypothalamus play a prominent role in regulating energy homeostasis. Future studies addressing how cilia influence neuronal activity through various mechanisms may reveal targets to treat this health concern.

### Human ciliopathies associated with obesity

Defects in cilia formation, structure, maintenance, and function are associated with a syndromic group of diseases called ciliopathies. Ciliopathies present with a wide variety of clinical features affecting nearly all tissues and organ systems. One shared clinical feature of several ciliopathies is childhood obesity (Table 1) (Reiter and Leroux, 2017).

**TABLE 1:** Obesity associated ciliopathies and genes. Several human ciliopathies share a common clinical feature, obesity. These include Bardet-Biedl syndrome, Alström syndrome, Carpenter syndrome, and MORM syndrome. Neuronal cilia also regulate energy homeostasis through different signaling pathways such as GPCR signaling and the Leptin-melanocortin Pathway (Seo et al., 2009; Obradovic et al., 2021; Yang et al., 2022b) that have proteins enriched along the cilia membrane. Additional cilia genes that are associated with obesity include CEP19, CEP290 (Leitch et al., 2008), MC4R, ADCY 3, and RPGRIP1L. Several of these cilia-enriched proteins show altered localization in ciliopathy models.

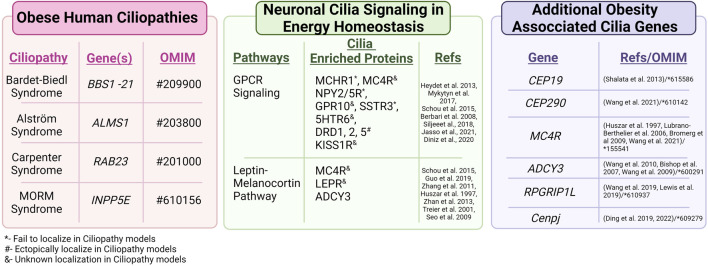

### Alström syndrome (ALMS, OMIM #203800)

ALMS was first characterized in 1959 as an autosomal recessive disorder that affects less than one out of one million individuals (Tsang et al., 2018). Unlike most ciliopathies, ALMS is associated with mutations in a single gene, *ALMS1*. ALMS1 protein localizes to the base of the cilium and is suggested to have a role in cilia formation and stability (Hearn et al., 2005). Interestingly, most human mutations of *ALMS1* lead to a truncated form of the protein that does not affect cilia formation (Hearn et al., 2005). However, these ALMS1 mutations inhibit proper cilia function and affect the long-term maintenance of the cilium (Hearn et al., 2002; Hearn et al., 2005; Knorz et al., 2010). Along with obesity, ALMS features severe insulin resistance associated with Type 2 diabetes mellitus, rod-cone dystrophy, cardiomyopathy, male infertility, and hearing loss (Mihai et al., 2008). ALMS patients do not necessarily show outward signs of neurological issues; however, brain abnormalities such as enlarged ventricles, grey and white matter atrophy, and diffuse supratentorial white matter derangement, demonstrates cilia play critical roles in the human CNS (Citton et al., 2013).

### Bardet-Biedl syndrome (BBS, OMIM #209900)

Similar to ALMS, BBS is another autosomal recessive disorder characterized by pediatric obesity; however, this syndrome is more common than ALMS, affecting around 1 in 100,000 individuals in North America and Europe (Forsythe et al., 2018). Many BBS patients have normal birth weight with obesity occurring in early childhood. Currently, more than 20 genes are implicated in cases of BBS, including BBS1-21 (Rohrschneider and Bolz, 2020), and most BBS proteins localize at cilia or near the base of the cilium. Eight of these gene products (BBS1, 2, 4, 5, 7, 8, 9, and 18) form the BBSome (Niederlova et al., 2019). The BBSome is a ciliary transport protein complex that mediates cargo trafficking to the base of, and removal from, the cilia membrane (Nachury et al., 2007; Jin and Nachury, 2009; Jin et al., 2010; Ye et al., 2018; Shinde et al., 2020). Different BBS mutations result in different degrees of obesity and other diagnostic phenotypes such as polydactyly, retinal degeneration, renal cysts, and hypogonadism (Forsythe et al., 2017; Niederlova et al., 2019). For example, mutations in BBS10 are more often associated with an earlier onset of obesity when compared to mutations in BBS1 (Pomeroy et al., 2021). Interestingly, BBS genes are important for the development of the central nervous system. For example, MRI scans of BBS patients show brain abnormalities including hypothalamic hypoplasia (Keppler-Noreuil et al., 2011), and empty sella of the pituitary (Soliman et al., 1996), both of which could contribute to growth and energy homeostasis features of the syndrome. Mouse models of BBS also show brain phenotypes like hippocampal hypoplasia and hydrocephalus (Bennouna-Greene et al., 2011; Singh et al., 2019).

### Other ciliopathies associated with obesity

Other rare disorders associated with cilia dysfunction and obesity are Carpenter and MORM Syndrome. Carpenter syndrome (CRPT1 OMIM #201000) is a developmental disorder characterized by obesity, craniofacial malformations, polysyndactyly, and intellectual disability (Hor et al., 2018). MORM syndrome (OMIM #610156) is classified by Mental retardation, Obesity, Retinal dystrophy, and Micropenis (Hakim et al., 2012). Homozygous null mutations in the Rab-GTPase, *RAB23*, lead to Carpenter syndrome. RAB23 protein is important for ciliary trafficking of receptors and proteins and is a negative regulator of hedgehog (HH) signaling. Mutations in the inositol polyphosphate-5-phosphatase E (*INPP5E*) gene lead to MORM syndrome (Zhang et al., 2022). INPP5E is a ciliary transition zone protein that is associated with establishing the different ciliary membrane compartments required to regulate signaling (Figure 1).

All of these syndromes are associated with mutations in genes important for cilia function, structure, and maintenance. Thus, cilia function is required for proper feeding behaviors and their dysfunction leads to obesity. However, the mechanisms that would link altered cilia function to obesity remain to be elucidated. It is likely that there are both developmental and adult homeostatic roles for cilia in energy homeostasis, and that cilia dysfunction impinges on their roles in the neuronal control of food intake and in peripheral tissues associated with energy homeostasis. Further studies to identify direct associations between ciliopathy gene function and energy homeostasis could identify potential therapeutic targets for these diseases.

### Clinical trials for ciliopathy associated obesity

Despite over 70% of BBS patients being overweight and obese, studies to elucidate the relationship between BBS genotypes and obese phenotypes have not revealed strong genotype-phenotype correlations (Pomeroy et al., 2021). This suggests that genetic background and the nature of the ciliopathy mutations and environment can play roles in the energy homeostasis phenotypes. Recently, one group used the Clinical Registry Investigating Bardet-Biedl Syndrome (CRIBBS) to look at the prevalence of obesity in BBS patients (Pomeroy et al., 2021). Children with BBS are often born with normal gestational length and weight but go on to show rapid weight gain and obesity in early childhood through adolescence. Loss of function variants in BBS genes are associated with a high risk for severe obesity (Pomeroy et al., 2021). Uncovering the specific genotypes of ciliopathy patients may help optimize the timing of potential therapies used to treat obesity. Previous trials for weight loss studies in Prader-Willi syndrome (PWS, OMIM #176270) used Methionine aminopeptidase 2 inhibitors (MetAP2i). These same inhibitors were shown to reduce hyperphagia in obese ciliopathy mouse models (Pottorf et al., 2020). While these studies showed reductions in weight and food intake in PWS patients, these trials have been suspended. More recently, a melanocortin-4 receptor (MC4R) agonist, Setmelanotide, has progressed to phase 3 trials (Clément et al., 2020). This drug is being tested to treat obesity in BBS and ALMS patients by reducing appetite and increasing energy expenditure (ClinicalTrials.gov, NCT04966741). Additionally, another clinical study is aimed at implementing caloric restriction in obese patients with autosomal dominant polycystic kidney disease (ADPKD, OMIM #173900), a disease characterized by mutations in cilia associated proteins polycystin-1 and -2 (Kim and Park, 2016). Here, the goal is to determine if weight loss can alter the progression of cystic disease (ClinicalTrials.gov, NCT04907799). Although these diseases are rare, development of both drug and behavioral interventions could be beneficial for ciliopathy patients. Ultimately, understanding how cilia are involved in energy homeostasis may also reveal novel targets for the general population of obese patients.

## Genetic mouse models of cilia associated obesity

Many genetic mouse models of obesity recapitulate phenotypes observed in humans, thus making them essential tools for studying the pathogenesis of obesity. A better understanding of cilia and their roles in obesity comes from mouse models of BBS and ALMS, as well as by mice carrying mutations in specific GPCRs known to localize to cilia and regulate energy homeostasis, such as melanin-concentrating hormone receptor 1 (MCHR1) and neuropeptide-Y 2 receptor (NPY2R). Additionally, mice with mutations in genes important for the formation and maintenance of primary cilia have proved essential to building our understanding of cilia regulated processes and pathways.

### BBS and ALMS mouse models are obese

Congenital mouse models of BBS and ALMS are obese, see (Vaisse et al., 2017) for BBS gene and obese mouse model elaboration. Two new obese mouse models for BBS include a gene trap allele (*Bbs5^−/−^*) thought to be a congenital null mutation and a conditional (*Bbs5^flox/flox^*) allele of *Bbs5*. Interestingly, the *Bbs5* conditional mutants become obese, independent of the age of Bbs5 loss implying homeostatic roles for *Bbs5* in energy homeostasis (Bentley-Ford et al., 2021; Bentley-Ford et al., 2022). Mouse models of ALMS have included a whole-body *Alms1* knockout (*Alms^flin/flin^*) (Geberhiwot et al., 2021) and the *fat aussie* mouse which carries a spontaneous 11 bp deletion resulting in a frameshift and truncating mutation in *Alms1* (Arsov et al., 2006; Kim et al., 2020). Both mice become obese and exhibit adipocyte hypertrophy, hyperglycemia, glucose intolerance, and leptin resistance. Interestingly, when *Alms1* was reintroduced to adipose tissue in the Almsflin/flin mouse, insulin sensitivity and glucose tolerance were restored (Geberhiwot et al., 2021). In *fat aussie*, Alms1 fails to localize to the base of the cilium in hypothalamic neurons and there is a significant reduction of adenylyl cyclase 3 (ADCY3) positive cilia (Heydet et al., 2013). It is unclear if the altered ciliary ADCY3 in this Alms1 model is due to loss of cilia or failure of ADCY3 to localize to the cilia membrane. This highlights a challenge for studies of neuronal cilia, the lack of a universal cilia marker in the central nervous system.

### GPCRs and neuronal cilia

There are several GPCRs enriched in neuronal cilia (Schou et al., 2015; Mykytyn and Askwith, 2017). If odorant and opsin receptors are included, then most GPCRs function at cilia. However, there is an emerging set of GPCRs that localize to cilia on neurons deep within the brain including MCHR1, melanocortin 4 receptor (MC4R), NPY2R, NPY5R, somatostatin receptor 3 (SSTR3), kisspeptin 1 receptor (KISS1R), serotonin receptor 6 (5HT6), and dopamine receptor 1 (DRD1) (Berbari et al., 2008a; Loktev and Jackson, 2013; Koemeter-Cox et al., 2014; Siljee et al., 2018) (Table 1). Many of these GPCRs have known roles in feeding behaviors, energy homeostasis, and altered cilia localization is observed in obese ciliopathy mouse models.

For instance, the MCH/MCHR1 signaling pathway has a well-defined role in energy homeostasis [for a review see (Al-Massadi et al., 2021)]. MCHR1 localizes to cilia in many areas of the mouse and rat brain, including the olfactory bulb, hippocampus, amygdala, hypothalamus, and spinal cord (Niño-Rivero et al., 2019; Diniz et al., 2020; Brewer et al., 2022). Using optogenetics, and chemogenetics, activation and inhibition of the MCH pathway causes cilia shortening and lengthening, respectively, in the brain as measured with ADCY3s staining (Alhassen et al., 2022). Interestingly, MCHR1 ligand, MCH, expression changes based on feeding status. For example, under fasted conditions, MCH increases in the lateral hypothalamus (Segal-Lieberman et al., 2003; Simon et al., 2018). There is also growing evidence to support the biological importance of ciliary GPCR signaling, such as MCHR1, in regulating cilia length and neuronal function (Miki et al., 2019; Kobayashi et al., 2020; Kobayashi et al., 2021). Ciliary MCHR1 may regulate different physiological conditions, such as feeding and be a potential target for conditions with impaired cilia function, such as ciliopathies. New mouse models, such as a fusion mCherry protein on the N-terminus of MCHR1 (Jasso et al., 2021) and an inducible *MCHR1* promoter driven cre allele (Engle et al., 2018), will aid in visual and additional functional analysis of this GPCR in the brain.

Many models of ciliopathies associated with obesity appear to mislocalize ciliary GPCRs. The BBSome plays an essential role in dynamically trafficking GPCRs to and from the cilia membrane [(Ye et al., 2018); (Nozaki et al., 2018; Nozaki et al., 2019; Zhou et al., 2022)]. BBS mouse models fail to localize MCHR1 and SSTR3 to the cilium in areas of the brain involved in feeding and reward pathways, such as the nucleus accumbens, olfactory bulb, and the hypothalamus (Berbari et al., 2008a). NPY2R and SSTR3 also fail to localize to the cilium in the absence of BBSome subunit BBIP10, a protein required for BBSome stability. These mice also fail to activate c-fos and decrease food intake in response to NPY2R ligand PYY3-36. Depletion of *Bbs3* showed normal NPY2R cilia localization (Loktev and Jackson, 2013); however, Bbs1 mutants have decreased NPY2R cilia localization and NPY2R expression, specifically in POMC and AgRP neurons (Guo et al., 2019). Interestingly, Bbs3 mutant mice have increased fat mass but do not develop overt obesity, and loss of Bbs3 allows for normal formation of the BBSome (Zhang et al., 2011). ALMS mouse models do not appear to mislocalize the GPCRs MCHR1 and SSTR3; however, *fat aussie* mouse models have a significant reduction in total cilia labeled with ADCY3 (Heydet et al., 2013). Together, ALMS and BBS may serve different functions in regulation of GPCR signaling in the cilium.

### MC4R signaling at the cilium

MC4R mutations compromise 3%–5% of cases of monogenic obesity in humans, making MC4R signaling and its downstream circuitry an appealing target for obesity therapeutics (Huszar et al., 1997; Lubrano-Berthelier et al., 2006; Bromberg et al., 2009). Neurons in the paraventricular nucleus of the hypothalamus (PVN) localize MC4R to cilia (Siljee et al., 2018). Loss of cilia in MC4R-expressing neurons causes obesity, hyperphagia, and increased body lengths (Siljee et al., 2018). AAV vector driven expression of normal and mutated forms of MC4R, localizes this GPCR with ADCY3 at the primary cilium (Siljee et al., 2018). This localization is significantly reduced in human MC4R mutants, p.P230L and p.R236C (Siljee et al., 2018). Interestingly, these mutations are found in the third intracellular loop of the GPCR, a region implicated in ciliary localization (Berbari et al., 2008b). Inhibition of ADCY3 activity at the cilia membrane using constitutively active Gα_i_ coupled GPR88 to inhibit MC4R at cilia caused mice to increase their food intake and become obese (Wang et al., 2020), demonstrating the importance of MC4R signaling at cilia.

Melanocortin receptors, like MC4R, require accessory proteins to regulate their activity and function (Rouault et al., 2017). For example, melanocortin receptor accessory protein 2 (MRAP2) plays a critical role in energy homeostasis in both mice and humans (Asai et al., 2013; Jackson et al., 2015; Bruschetta et al., 2018). Additionally, MC4R and MRAP2 are co-expressed in many cells within the PVN (Asai et al., 2013). Mice with global null mutations of MRAP2 are significantly heavier than littermate controls. MRAP2 deletion specifically in Sim1-expressing neurons of the PVN causes obesity in mice (Asai et al., 2013). MRAP2 and MC4R interact directly with each other to enhance MC4R stimulated cyclic adenosine monophosphate (cAMP) production (Asai et al., 2013; Jackson et al., 2015). Recent data also shows that MRAP2 promotes the cilia localization of MC4R (Bernard et al., 2020). This raises the question if other neuronal GPCRs, such as MCHR1 and NPY2R, are regulated by MRAPs or other accessory proteins. Understanding the molecular mechanisms of neuronal GPCR localization and function will aid in the development of drug treatments for metabolic disorders.

### Altered cilia signaling machinery and obesity

Although GPCR signaling is a common paradigm that cilia use to regulate different physiological processes like vision and olfaction, it is emerging that cilia may utilize other signaling mechanisms in the brain. For example, a recent study has proposed the idea of axo-ciliary synapses. Here, they show that artificial stimulation of serotonergic axons releases serotonin directly onto the ciliary receptor 5-hydroxytrptamine receptor 6 (5-HTR6) in hippocampal neurons to activate the Gα_q/11_ RhoA pathway (Sheu et al., 2022). Further research could elucidate other neuronal populations that may use their cilium to form axo-ciliary synapses to regulate different functions, perhaps those involved in energy homeostasis.

Other alternate mechanisms for ciliary signaling could involve downstream effectors of GPCRs such as ADCY3. Polymorphisms in ADCY3 are associated with obesity in humans (Nordman et al., 2008; Wang et al., 2010). ADCY3 is highly expressed in the hypothalamus and shown to localize to neuronal primary cilia (Bishop et al., 2007; Domire and Mykytyn, 2009; Caspary et al., 2016). Mice lacking ADCY3 exhibit obesity that is caused by a decrease in activity, hyperphagia, and leptin resistance. Additionally, ADCY3 activity in the hypothalamus was reduced upon forskolin stimulation (Wang et al., 2009). Similar results are observed in male and female mice using an AAV-CRE GFP injection into the hypothalamus of conditional ADCY3 animals (Cao et al., 2016). These data point toward a role for hypothalamic ADCY3 in regulating feeding behaviors in both mice and humans.

Furthermore, when ADCY3 deletion is specific to the ventromedial hypothalamus (VMH), weight gain is pronounced in animals on a high fat diet (HFD). In addition, ADCY3 regulates autophagy by binding to gamma-aminobutyric acid A receptor-associated protein (GABARAP) (Yang et al., 2022a). Interestingly, autophagy is another mechanism thought to be used by cilia to regulate signaling (Pampliega et al., 2013; Orhon et al., 2015), and there is growing evidence to support a bi-directional relationship between ciliogenesis and autophagy (Ávalos et al., 2017). In ADCY3 knockout mice, there is a reduction of p62 and an increase in LC3-II, two proteins that regulate autophagy (Cao et al., 2016). Similarly, an increase in LC3-II and decrease in p62 is observed when overexpression of ADCY3 is inhibited using an AVV carrying constitutively active GPR88, a Gi- protein coupled receptor ((Siljee et al., 2018); (Yang et al., 2022a)).

High fat diets are rich in saturated fatty acids, mainly palmitic acid, and known to be the main cause of visceral obesity, glucose intolerance, and insulin resistance (Tchernof and Després, 2013). Palmitic acid is significantly increased in the hypothalamus of mice under chronic HFD conditions and in the plasma of obese humans (Kang et al., 2017). Additionally, chronic HFD in mice decreases the number and length of cilia of POMC neurons (Ávalos et al., 2022). Interestingly, treatment of hypothalamic neurons with palmitic acid impairs autophagy [(Hernández-Cáceres et al., 2019); (Hernández-Cáceres et al., 2020)] and reduces cilia number and length and blocks insulin-dependent signaling [(Ávalos et al., 2022); (Hernández-Cáceres et al., 2020)]. In developing POMC neurons, removal of Intraflagellar Transport 88 (IFT88) or kinesin family member 3A (Kif3A) disrupts axonal projections from the ARC to the PVN and development of POMC neurons through impaired lysosome protein degradation [(Ding et al., 2021); (Croizier and Bouret, 2022)]. Together, these results show that autophagy may regulate ciliary signaling and the proper localization of cilia proteins. Body composition and diet influences autophagy mechanisms important for ciliogenesis and cilia signaling as well as protein degradation in a cilia dependent manner.

### Transition zone and basal body in energy homeostasis

The cilium regulates its structure maintenance and extracellular signaling processing through different compartments along its axoneme, such as at the transition zone and basal body. Several centrosome related gene mutations are also associated with obesity. Ciliopathies such as ALMS and BBS have mutations in genes associated with the centrosome. *CEP19* is a cilia and centrosome associated protein that is highly conserved in vertebrates and invertebrates. Cep-19 knockout mice are morbidly obese, hyperphagic, glucose intolerant, and insulin resistant which recapitulates *CEP19* mutations in humans (Shalata et al., 2013) (OMIM #615586). Centromere protein J (Cenpj) is a protein crucial for centrosome biogenesis and elongation, cilium disassembly, and spindle pole integrity. Depletion of Cenpj results in long cilia and abnormal cilia disassembly in neural progenitor cells *in vivo* (Ding et al., 2019). Conditional knockout of Cenpj in the hypothalamus results in decreased proliferation and increased apoptosis during embryonic development (Ding et al., 2021). These mice became obese, hyperphagic and less active in adulthood.

The Abelson-helper integration site 1 (*Ahi1*) gene product is required for localizing proteins to the transition zone of the cilium. When mutated, this gene causes the human ciliopathy Joubert Syndrome (JBTS, OMIM #213300) which does not routinely present with obesity [(Thomas et al., 2015); (Adams et al., 2007)]. In Ahi1 mutant mice, MCHR1 expression in neurons was similar compared to littermates; however, ciliary localization of MCHR1 was significantly reduced. Ahi1 depletion also led to the downregulation of two downstream signaling pathways of MCHR1, cAMP and extracellular signal-regulated kinase (ERK), upon ligand stimulation (Hsiao et al., 2021). Further evidence would be required to see how Ahi1 associated MCHR1 mislocalization impacts physiological processes, such as feeding. Another cilia transition zone protein, retinitis pigmentosa GTPase regulator-interacting protein-1 like (RPGRIP1L), is also implicated in feeding, as conditional ablation of RPGRIP1L leads to obesity in mice [(Lewis et al., 2019); (Wang et al., 2019)]. Congenital RPGRIP1L hypomorphism in POMC neurons leads to hyperphagic obesity and increased adiposity; however, deletion of RPGRIP1L in adult POMC neurons did not result in an obesity phenotype. These studies also report a reduction in the ratio of POMC and Neuropeptide-Y (NPY) neurons with an increase in axonal projections between the arcuate nucleus of the hypothalamus (ARC) and PVN. These findings suggest that hypothalamic RPGRIP1L polymorphisms impact the development of POMC neurons and their derivatives (Wang et al., 2019).

## Neuronal cilia populations and feeding behaviors

**FIGURE 2:**
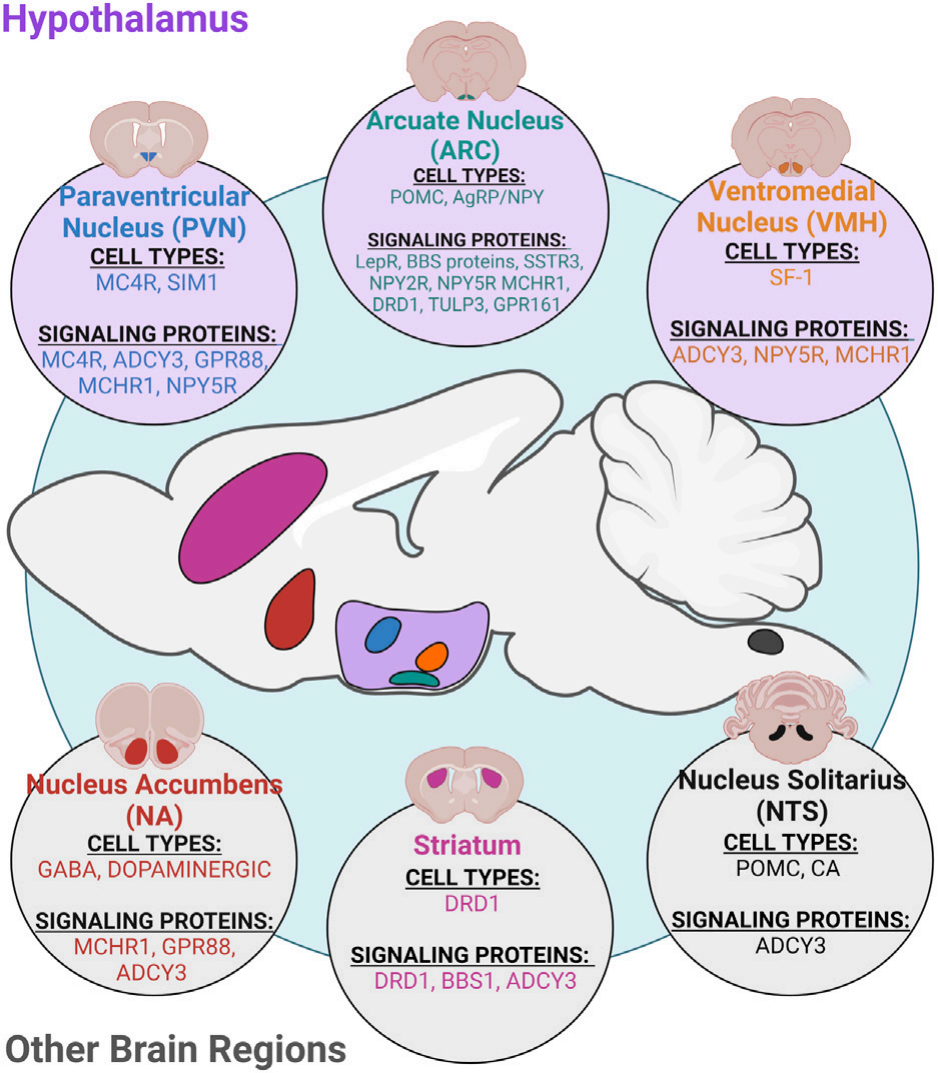
Ciliary neuronal populations in the CNS. Primary cilia are found on most neuronal cell types in the CNS and play roles in regulating different physiological processes. Specifically, cilia on different cell types in the hypothalamus and other brain regions such as the nucleus accumbens (NA), striatum, and nucleus solitarius (NTS) have known roles in regulating energy homeostasis and feeding. Cilia on hypothalamic neurons in the paraventricular nucleus (PVN), arcuate nucleus (ARC), and ventromedial nucleus (VMH) have roles in regulating feeding and metabolism (Fetissov et al., 2004; Nguyen et al., 2012; Chávez et al., 2015; Ehrlich et al., 2018; Nishimura et al., 2019; Nyamugenda et al., 2019; Ramos et al., 2021).

The brain is vital for integrating and coordinating signals, such as hormones and nutrients, to maintain energy homeostasis. Cilia on neurons are required for normal energy homeostasis as conditional knockout models of ciliogenesis genes, IFT88 and Kif3A, cause obesity (Davenport et al., 2007; Lechtreck, 2015; Lee et al., 2020). Here, we discuss the roles of neuronal cilia in the different nuclei of the hypothalamus and the localization of GPCRs, specifically MC4R, to hypothalamic neuronal cilia in response to feeding. Many of these recent data suggest that neuronal cilia of the hypothalamus may work together to create a metabolic signaling hub critical for proper energy homeostasis (Figure 2).

### Conditional cilia models implicating the hypothalamus

The hypothalamus is a key region in the brain known to regulate feeding behaviors and metabolism, and it consists of many well-defined nuclei, including the ventromedial hypothalamus (VMH), arcuate nucleus (ARC), and paraventricular nucleus (PVN) (Figure 2). The VMH is important for many homeostatic processes including, skeletal homeostasis, mood behavior, reproduction, and regulation of body weight and glucose homeostasis (Krause and Ingraham, 2017). Primary cilia in the VMH are longer than other neuronal cilia types (Sipos et al., 2018) which are significantly shorter under metabolic conditions such as obesity and leptin resistance (Han et al., 2014). Genetic ablation of cilia using Cre-loxP and bilateral AAV-Cre injection in VMH steroidogenic factor-1 (SF1) expressing neurons caused an increase in body weight, subcutaneous and gonadal adipose tissue increase, and elevated serum leptin levels (Sun et al., 2021). Additionally, food intake was significantly increased in VMH cilia mutants, and they showed reduced activity, especially during the dark cycle, and reduced brown adipose tissue thermogenesis (Sun et al., 2021). Interestingly, humanized ACDCY3 knock-in mice are resistant to high-fat diet induced obesity and show an increase in cilia frequency and length in the VMH. Injection of ciliary ADCY3 inhibitor GPR88 (Siljee et al., 2018) resulted in obesity under normal chow and attenuated weight gain under HFD. Similarly, Bbs1 deletion in the VMH using a SIM1 cre approach results in obesity without altering food intake, energy absorption, and digestive efficiency (Rouabhi et al., 2021).

Conditional knockout of IFT88 or Kif3A in neonatal POMC-expressing neurons leads to obesity in adult mice (Davenport et al., 2007). Interestingly, conditional ablation of cilia in adult POMC neurons did not result in significant changes in body weight, food intake, or energy expenditure suggesting that cilia on these neurons are important for their embryonic development and early post-natal circuit organization (Wang et al., 2019). Deletion of *Bbs1* in POMC or AgRP neurons disrupts the formation of the BBSome and increases body weight and adiposity. Specifically, this obesity in driven by hyperphagia when the BBSome is disrupted in POMC neurons, and these mice were glucose intolerant and insulin resistant. In AgRP neurons, disruption of the BBSome shows a more pronounced weight gain and increase in fat mass in females compared to males (Guo et al., 2019). It is clear that proper cilia assembly and signaling in the ARC are important for regulating energy balance. Cenpj conditional knockout models lead to hypothalamic defects early on and reduced area size of the ARC and PVN in adults. Additionally, depletion of Cenpj leads to a decrease in POMC neurons, a reduction of POMC neuronal projections into the PVN, and increased spontaneous firing of NPY neurons in the ARC (Ding et al., 2021). It appears that an increase in AgRP/NPY and decrease in alpha melano-stimulating hormone (α-MSH) blocks satiety causing the obese phenotype (Ding et al., 2021).

### Cilia in other brain regions associated with energy homeostasis

Although the hypothalamus appears to be the main brain region for neuronal cilia regulation of feeding and metabolism, cilia in other regions in the CNS may contribute to energy homeostasis (Figure 2). For example, in Bbs1 mutant mice, dopamine receptor 1 (DRD1), localizes to the cilium in the striatum, amygdala, and olfactory tubercle (Domire et al., 2011). Interestingly, in BBS mutants, DRD1 localization to the cilium is accompanied by a reduction of ciliary ADCY3 in DRD1-expressing neurons. These mice become obese as a result of reduced locomotor activity which is recapitulated in DRD1 cilia knockout mice (Stubbs et al., 2022). Additionally, the hindbrain includes regions such as the nucleus tractus solitarius (NTS) that have implicated roles in feeding behavior. Here, POMC neurons located in the NTS respond to short term satiety signals in the brain stem to regulate energy homeostasis, whereas POMC neurons in the ARC respond to long-term feeding signals [(Cheng et al., 2021); (Zhan et al., 2013)]. When cilia are ablated from POMC neurons, through Kif3A, mice are obese and present with an increase in adiposity, lean mass, and body length (Davenport et al., 2007). Although this study focused on POMC neurons in the hypothalamus, it is possible that cilia ablation on POMC neurons in the NTS could contribute to the obese phenotype. These data suggest that cilia in other areas of the brain may also regulate energy homeostasis through additional mechanisms, such as influencing locomotion or at specific time points. Future studies should expand upon understanding the role for cilia in these different brain regions.

## Neurodevelopmental roles for cilia in obesity

The most well-defined roles for primary cilia come from our understanding of how they mediate hedgehog signaling in embryonic development and tissue patterning [for a review see (Goetz and Anderson, 2010)]. Cilia-mediated hedgehog signaling is critical for patterning many tissues, including the developing hypothalamus (Szabó et al., 2009; Shimogori et al., 2010; Blaess et al., 2014) and pituitary (Treier et al., 2001). In mouse models of BBS, mispatterning of the hypothalamus is associated with a potential loss of 20% of POMC neurons in BBS2 and BBS6 mutants (Seo et al., 2009). In cultured BBS mutant iPSC cells, hedgehog signaling plays a role in differentiation into arcuate and other hypothalamic neuronal fates (Wang et al., 2019; Wang et al., 2021b). In addition, pituitary phenotypes consistent with perturbations in Hedgehog patterning defects occur in a mouse model of BBS5 (Bentley-Ford et al., 2021). These observations indicate the potential for altered hedgehog-mediated patterning to contribute to ciliopathy-associated obesity later in life.

## Challenges and future directions and conclusions

There are many challenges to understanding neuronal cilia. Simply visualizing cilia in the CNS is a challenge, as the standard tubulin markers are not specific to the organelle in neurons (Caspary et al., 2016). Even fundamental questions around understanding if neuronal cilia act through slower neuropeptide mediated mechanisms or directly influence neuronal activity, as suggested with the observation that cilia can directly synapse to neurons (Sheu et al., 2022), or perhaps cilia serve as both slow and fast modulators of neurons and circuits. Fascinating work in models like *C. elegans* has suggested that neuronal cilia length changes can directly impact their ability to sense the external environment (Maurya and Sengupta, 2022). In addition, neuronal cilia also appear to be capable of sending signals not just receiving them pointing to the complexity of neuronal cilia signaling [(Wang et al., 2021c); (Nikonorova et al., 2022)]. In conclusion, how cilia in the brain regulate energy homeostasis has become a complex question with impacts beyond the field of obesity and into general neuroscience and signaling. It is likely that both developmental and homeostatic processes regulated by CNS cilia can contribute to obesity. It is also likely that multiple pathways are perturbed in the context of ciliopathies leading to hyperphagia and obesity.
